# A General Outline of the Treatment of Eczema

**Published:** 1890-06

**Authors:** George T. Elliott

**Affiliations:** Dermatologist to Demilt Dispensary and the New York Infant Asylum; Assistant Physician New York Skin and Cancer Hospital, etc.


VOL. VII. JUNE, 1890. No. 4.

### Original Communications.

### A GENERAL OUTLINE OF THE TREATMENT OF ECZEMA.

BY GEORGE T. ELLIOT, M. D.,

Dermatologist to Demilt Dispensary and the New York Infant Asylum; Assistant Physician New York Skin and Cancer Hospital, etc.

The treatment of eczema constitutes such an enormous subject and covers so broad a field, that to enter upon it in a particular manner and to consider it in all its details would require so much time and would make so great demands upon your patience, that I have thought it preferable to give only a general outline of the most important procedures and the most serviceable remedies, and to confine myself to those major points, which will furnish a working basis and enable the therapeutic care of any case to be directed and carried out in a proper manner. In consequence, I propose to deal only with the general indications for the treatment of eczema—the internal medication necessary and the external applications which have, in my experience, proved most useful, because it is impossible to give all of those which are recommended or used. Before taking up any one of these propositions, however, I would refer briefly to the etiological and pathological factors which are concerned in the production of eczema, inasmuch as their recognition is indispensable, both for the comprehension of the disease, and also for the choice of the line of treatment to be followed and of the remedies to be exhibited. And I would do this likewise, on account of the tendency there is among those who are not specialists, to regard eczema as a morbid entity, always the outcome, or the result, or the expression of some one cause, which cause is to be attributed to and sought for in some particular pathological state of the general system, in a dyscrasia, a diathesis, or in that most indefinitely and deplorably termed condition—impurity of the blood. As a result of such a tendency, the basis of the disease is usually misunderstood, its essential features are lost sight of, no attempt is made to seek out the exciting or determining cause in any given case, but the treatment is instituted without reference to the etiological or pathological factors, which have existed and are still existing; in other words, a routine and stock treatment is followed in every case, which, occasionally beneficial, is, as a rule, entirely inefficacious and useless. It has, for instance, repeatedly occurred to me to see patients with marked gouty symptoms receiving for their eczema stimulants, tonics, a full nitrogenous diet and an oxide of zinc ointment, and, on the other hand, a child, with the disease caused by *pediculi capitis*, taking arsenic for weeks and months, no attention being paid to the *fons et origo mali*—the *pediculi*—and no attempt being made to get rid of them. Such occurrences should not and would not be observed, however, if the pathological bases of the disease were properly considered or understood, and, if it was borne in mind that eczema does not owe its origin to any one and single but to innumerable causes, which may be systemic or local in nature, or the disease may also be due to the combined action of both an internal and an external cause. An eczema purely of internal causation is, comparatively speaking, infrequently met with; much more commonly the disease owes its origin to some systemic disturbance acting in co-operation with a local irritant; possibly the most numerous cases are those of external production alone. The effect of a pure internal disturbance determining an outbreak of eczema is seen in the occurrence of the disease after severe nervous shocks and neuralgic attacks, but more usually it is met with as a result of irritation in some distant portion of the general system, that is, it appears in the form of a reflex neurosis. Such eczemas are frequently seen in babies and in children suffering from a gastric or an intestinal catarrh, or intestinal worms, etc.; they constitute also the so-called dentition eczemas, and I have even seen it develop in babies under the influence of the general irritation due to a cystitis. In women, also, eczema occurs as a reflex neurosis, starting from some uterine disorder. Certain ones of these disturbances do not, however, act always and alone in a reflex manner, but very frequently in the same way as do other systemic conditions and diseases, functional or organic, which serve only as predisposing and contributive causes in the production of eczema. The influence exerted by these is due to the fact that they impair the nutrition of the skin as well as of the general system; they increase its irritability and diminish and reduce its natural resistive power and capability of withstanding attacks and irritation from the external influences to which the cutaneous surface is continually exposed. As examples of these predisposing causes, there can be named gout, rheumatism in all of its forms, Bright's disease, diabetes, chronic constipation, dyspepsia, and mal-assimilation. Anæmia and plethora are included here, and also a low power of oxidation and a want of proper balance between elimination and waste, as is so frequently observed in elderly people. In a more local form the contributive factor may be found in the congestion and œdema of the legs produced by a varicose condition of the veins. The presence of one or more of these systemic conditions does not, however directly, and in virtue of it and themselves, cause the appearance of an eczema, but for that purpose there is required an external influence of some kind, which represents the determining and exciting factor. This latter may be of only the very slightest nature, such as scratching, rubbing, or the accumulation and decomposition of the natural secretions of the skin in certain localities—under the hanging breasts in women; in the inguino-scrotal region, etc.; or again it may be constituted by any one of the very numerous agents and substances, which, owing to their own inherent and irritating properties, are able to produce an eczema upon an individual in every way healthy. The number of these substances is practically without limit, but I need only cite a few of these, such as those with which a person is brought into intimate and constant contact from the nature of his occupation or work, as is the case with electro-platers, workers in oils, chemicals, acids, dye-stuffs, with marble cutters, etc., etc. Certain drugs also possess the property of causing the disease, as arnica, mercuric chloride, iodoform, turpentine, the use of sulphur, and mercurial inunctions. Again, the exciting cause may be the dye from a poor quality of stockings, or the too free use of water in the form of baths, or in those doing house and laundry work, the neglect to dry the hands properly, the use of a soap containing an excess of free alkali, or of such cleansing agents as pearline, soapine. Heat and cold may determine an eczema, and also vegetable parasites, the scratching induced by the bites of animal parasites, by a pruritus, an urticaria, or other itchy condition of the skin.

This brief review of the factors which are productive of eczema will serve to point out how diverse and innumerable are the causes of the cutaneous disease and their consideration cannot but show that under the circumstances, there is no room for routine in its treatment. On the contrary, for this latter to be successful, not only must the exciting and predisposing factors which have been in operation in any given case be carefully valued ; but likewise all other indications must be properly met, and the treatment will have to be varied in accordance with the symptoms and requirements in each example of the disease. Of course it will not be possible to trace out the exact determining factor in every case of eczema, but nevertheless it may be laid down as a general and primary rule in the treatment of the disease, that its cause, both exciting and predisposi[illegible], must be diligently sought for and the endeavor should also be [illegible]

to determine in which class any particular case belongs ; that is, whether it is of internal origin alone, or simply a local outbreak or due to the participation of both factors. They are the key-note upon which depend the therapeutic measures to be instituted and the result obtained. Therefore a careful investigation of the patient's hereditary antecedents, his history previous to his attack, the condition of the gastro-intestinal canal, liver and other organs should be made. In the elderly, the miscroscopic examination of the urine is very necessary. A general view of the patient's mode of life should be obtained ; his habits, diet and hygienic surroundings ought to be inquired into ; the clothing worn, the work or occupation and the substances with which these bring him into contact must receive attention ; his physical condition and development, the presence of any systemic disease or pathological state should also be determined; in other words a thorough comprehension of everything concerning the patient should be sought for and as far as possible obtained. It will naturally not be necessary to enter into all the minutiæ in every case, the line of investigation demanded being quickly suggested by the age of the patient, his station in life and the familiarity of the physician with disease in general. Still a wide-reaching examination will oftentimes be called for, owing to the pertinacity and rebelliousness of an eczema, its constant relapsing and resistance to all treatment applied. A child, for instance, who goes to a public school has an eczema of the scalp, and to a slight extent of the face and the ears. The first impulse will be to look for pediculi or their nits, since they are a very common cause of eczema of the scalp in school children. On the other hand, the patient may be an elderly man in good position, who has had for years perhaps a very pruritic eczema, about the flexures of the joints especially. In such a case, the possibility of the eruption being dependent upon some one or other systemic change or disease, will demand a careful and extensive investigation. The patient, however, having been thoroughly examined and all the features of the case noted, the treatment which is instituted must have for its object the removal of every possible predisposing and exciting factor, and the placing of the patient in the most favorable condition of health.

To attain this end, rectify every deviation from the normal standard, regulate faulty diet and hygiene, enjoin cleanliness, proper frequency in changing and suitable clothing (a baby will frequently develop an eczema owing to soiled and wet diapers being allowed to remain in contact with the skin), warn against the excessive use of water in any form, and recommend avoidance of all irritating agents or substances, whether medicinal, chemical, or of other nature. If it is an eczema due to the trade the patient has, precautions for protecting the exposed surfaces must be taken, or it may be advisable for him to give up for a time his particular kind of work, or it may become imperative for him to change his occupation entirely. These general matters having been attended to, the therapeutical part of the treatment may now be undertaken. It consists of the internal remedies, which will be indicated by the systemic disturbances present, and of the external applications, which act more directly upon the diseased surfaces.

As far as the former are concerned, they are in every way the same remedies as would be given were there no eczema present, but only some systemic disturbance or other. There are no specifics for eczema, no drugs which influence the pathological process directly, and in virtue of their own peculiar properties or action, but the benefit which is obtained from the use of any medicinal agent internally is through its power or capability of removing or improving those functional or other disturbances of health, which brought about a predisposing and favorable condition for the development of the disease. The internal treatment of eczema can be summed up by describing it as a purely symptomatic one, depending upon and varying according to the indications present and having for its object the remedying of each and every defect which may exist. It may, however, be said that, in acute cases, saline purgatives are specially useful. In the chronic forms, when there is much turgescence of the skin, an occasional dose of Epsom or Rochelle salts will also give considerable relief, but I have never, however, seen any good result from the exhibition of salts in small doses for a long time. When constipation has been present, I have given according to the indications in each case, rhubarb or cascara or calomel, or in the gouty, Carlsbad salts, with equally good results. For babies magnesia, but especially castor oil, is advisable. Should a gastric or intestinal catarrh or dyspepsia be present, then digestives, bitters, anti-fermentatives, intestinal antiseptics, sterilization of milk, etc., are as much indicated as when uncomplicated by an eczema on the skin. In anæmia, give iron and other tonic remedies ; in tubercular subjects, fats, cod-liver oil, etc.; in neurotic cases, remove the cause operating in a reflex manner and administer nerve sedatives, tonics, etc.; in babies irritable from dentition, a few grains of some one of the bromides will often aid in quieting the irritation and enable a troublesome eczema to be controlled, and eventually appropriate local applications will enable a cure to be obtained.

The use of the alkalies, the potash salts, especially the lithia compounds, etc., in eczema is so general and extensive that I may be permitted to devote attention to them a little more particularly. Their administration should, however, be guided by the same rule that governs other remedies—that is, they should be given when demanded by the systemic symptoms. They will then be of great value and productive of inestimable good, but under other circumstances it is ammunition thrown away. In babies and children, it has happened to me not infrequently to find the urine containing more or less large quantities of uric acid and oxalate of lime, and in such cases the liq. potassa in appropriate doses often proves very serviceable. In older children, a lithiated or alkaline mineral water will often be sufficient. When there is an excess of acid in the stomach, bicarbonate of sodium is a reliable remedy, or lime water will at times be all that is necessary.

But it is for adults that the alkaline remedies are more particularly indicated, especially among the better class of patients, whose mode of life predisposes them to gout, and to the accumulation of waste products in the system, and likewise for elderly people, with low oxidizing and deficient powers of elimination. In these the citrate, acetate or bicarbonate of potash and the lithia compounds are of great benefit, especially if free diuresis is provoked by drinking a good deal of water. These alkalies may be used of course alone, or in combination with any other remedy or remedies indicated ; in gout, with colchicum, or in anæmia with iron, when there is deficient cardiac power, with digitalis, and so on.

There is another drug also which is usually looked upon as a specific for eczema, and which has obtained for itself a foremost position, and that is—arsenic. By what means it has arrived at this point, it would be difficult to say, as its influence has been and is greatly overrated, and when it is productive of good it is so only in certain classes of cases. Even in these a cure can be obtained without it, and I may say that though treating yearly from four to five hundred cases of eczema in all of its forms, I am certain that I do not prescribe arsenic for more than twenty or thirty of them. In the acute stages of the disease it is contra-indicated, and likewise when there is much inflammation present, or when, the skin is irritable and becomes very itchy while the drug is being taken ; it is also practically valueless and often harmful in weeping and pustular, in trade eczemas, and in those due entirely to local irritation.

In chronic forms of the disease, when there are no evidences of active inflammation, a torpid condition of the skin and considerable infiltration, when they are of the papular and squamous variety, arsenic, however, is frequently very serviceable. In tubercular children, with lamphomata of the neck, who are pale and pasty, weak and flabby, and have suffered from a squamous and infiltrated or papular eczema for years, it is often very useful, but the drug alone will not produce much of a result, unless it is combined with the other measures indicated, whether they be tonic or dietetic or hygienic, etc. There is no particular choice between the several preparations of arsenic. Fowler's or Donovan's solution or the liq. sodæ arseniatis may be used, or the arsenious acid may be given in pill form. The dose should be at first small and then increased quite rapidly to a safe point, at which it must be maintained. Beginning for instance with nine drops of Fowler's daily, an additional one drop may be added every other day, until the patient is taking twenty or thirty or more drops a day with perfect safety. In giving arsenic, however, it must be remembered that a most severe and intense pruritus may be produced by it, and also that its use is sometimes followed by an extensive brown pigmentation of the skin.

The diet in eczema is of considerable importance. It will vary according to the necessities of each patient and the systemic disturbances from which he is suffering, as in gouty, rheumatic, diabetic subjects, but in general fermentable articles should be prohibited, sugar, candy and sweets allowed only in moderation. Highly seasoned and made dishes, salt meats, stimulating articles, pepper, mustard and condiments be either entirely forbidden or permitted to be taken only occasionally. The diet should be plain and nutritious, composed of easily digested and assimilable food, and suited to the age of the patient. Both the quality and the quantity of the milk taken by babies should receive particular attention, and whatever defect exists must be remedied. Often a baby will be overfed, or receive improper articles of food; in such cases stringent and rigid regulations of the diet must be instituted. Oftentimes change from the mother's to cow's milk will be necessary, or the amount taken by the baby must be supplemented by some of the prepared foods. Weaning the baby is especially advisable when the mother is suffering from any general or local disease which materially affects her health. I can recall to mind a baby who was under my care some time ago for an extensive eczema of the face, arms and trunk. The mother had an abscess of the breast, but nevertheless absolutely insisted on nursing the baby, even on the inflamed and suppurating gland. All treatment was of course without effect, and not until some two months later, and after the mother had entirely recovered her health, was it possible to do anything for the child. Similar cases occur every day, and it is also a common matter of observation to see babies with eczema get quickly well when their diet is properly regulated. Consequently, particular care is this direction is strongly advised.

Alcohol need not be entirely forbidden to patients with eczema, but the quantity taken should be reduced to a minimum. Beer and strong wines must be prohibited. Tea and coffee in my experience do not act injuriously when taken in moderate quantities, except in certain patients, but their excessive use should be restricted in every case. The internal treatment may thus be said to be directed entirely towards removing any and all deviations from health, and when properly carried out will alone not infrequently lead to complete involution of the disease, since by its means the skin loses its vulnerability or irritability and no longer reacts so easily to an irritation or other external and determining factor.

(*To be concluded next issue.*)

